# Exploring shape changes in healthy bone growth through 3D spatiotemporal statistical shape models: A scoping review

**DOI:** 10.1016/j.bonr.2024.101817

**Published:** 2024-11-22

**Authors:** Lily E. de Vries, Derek F.R. van Loon, Eline M. van Es, DirkJan H.E.J. Veeger, Joost W. Colaris

**Affiliations:** a Erasmus MC, University Medical Center Rotterdam, Department of Orthopedics and Sports Medicine, 3000 CA Rotterdam, the Netherlands; b Educational program Technical Medicine, Leiden University Medical Center, Delft University of Technology, Erasmus University Medical Center Rotterdam, the Netherlands; c Department of Biomechanical Engineering, Delft University of Technology, 2628 CD Delft, the Netherlands

**Keywords:** Statistical shape model, Bone growth, Three-dimensional imaging, Morphological model, Patient-specific model

## Abstract

**Objective:**

Analyzing population trends of bone shape variation can provide valuable insights into growth processes. This review aims to overview state-of-the-art spatiotemporal statistical shape modeling techniques, emphasizing their application to 3D skeletal structures during healthy growth.

**Methods:**

We searched PubMed and Scopus for articles on statistical shape modeling using a pediatric spatiotemporal dataset of 3D healthy bone models. Dataset characteristics and details on the shape models' development, analyses, and potential clinical use were extracted.

**Results:**

Fourteen studies were found eligible, modeling one or multiple lower limb bones, the mandible, the skull, and vertebrae. The majority applied Principal Component Analysis on point distribution models to create a statistical shape model. Shape variation was analyzed based on shape modes, representing a specific shape change as a part of the overall variance. Unscaled models resulted in a more compact statistical shape model than scaled models. The latter represented more subtle shape variations due to the absence of size differences between the bone models. Four studies reported a significant correlation between the first shape mode and age, indicating a relationship between that type of shape variation and growth. Three studies reconstructed 3D models using prediction features of statistical shape modeling. Measuring difference between predicted and actual anatomy resulted in Root Mean Squared Errors below 3 mm.

**Conclusion:**

Spatiotemporal statistical shape modeling provides insight into modes of shape variation during growth. Such a model can be used to find predictive factors, like age or sex, and deploy these characteristics to predict someone's bone geometry.

## Highlights

• Spatiotemporal statistical shape modeling can capture and visualize shape changes in bone anatomy during healthy growth.
• Principal Component Analysis and Partial Least Squares Regression are common techniques for statistical shape modeling.
• Statistical shape modeling enables the prediction of skeletal geometry based on personal characteristics.
• Spatiotemporal statistical shape modeling could be valuable for diagnosing and treating orthopedic deformities.

## 1 Introduction

Growth is the dominant cause of bone change during childhood. Clinicians cannot distinguish healthy from pathological deviations without insight into regular anatomical changes during growth. In orthopedics, average growth of long bones is often captured in bone length measurements and closure of growth plates. These measurements are usually based on 2D imaging, while bones have three-dimensional (3D) shapes. Statistical shape models (SSMs), representing the population's anatomy in 3D have proven useful in helping understand such normal variations in anatomy (Ambellan et al., 2019).

**Fig. 1:**
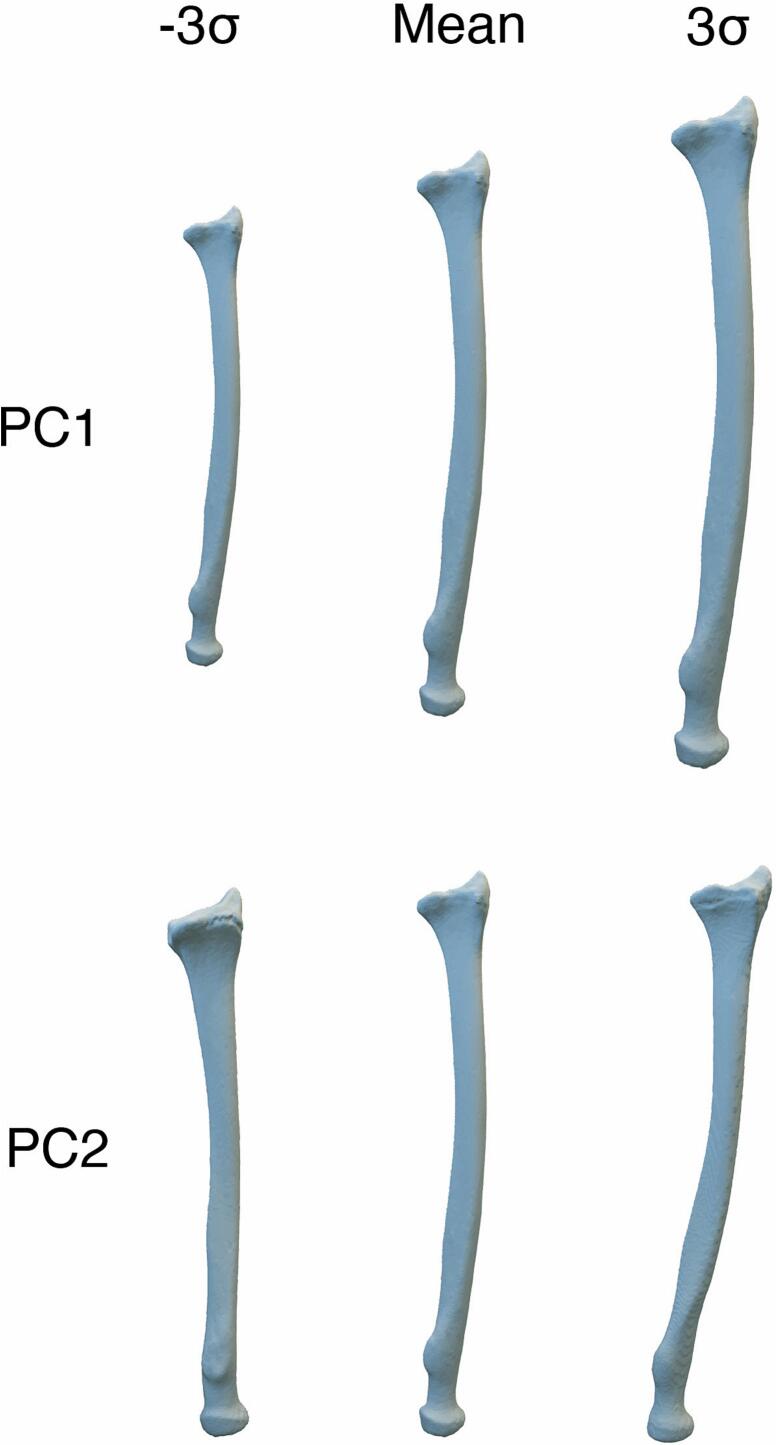
The first two shape modes for a radius shape model. Each row shows the variation within a principal component between -3σ and + 3σ. In this example, PC1 captures a difference in overall size. PC2 represents variation in angulation in the coronal plane.

SSMs are a mathematical representation of common shape variability within a dataset of structures, like bones (Cootes et al., 1992). Given a particular shape (such as a radius in Fig. 1), Principal Component Analysis (PCA) provides insight into a dataset's different types of shape variation, known as shape modes or principal components (PC). Each mode corresponds to a particular kind of variation and provides an axis along which shapes vary the most from the dataset's mean shape.

Using a cross-sectional dataset of pediatric bones at different ages, certain shape variations might be linked to age and, thus growth. These models are called spatiotemporal SSMs, as shapes at different time points are analyzed. A shape variation related to an increase in age can be explained as the average growth. By incorporating more demographic characteristics, such as sex or ethnic origin, the mean growth trajectory for specific groups can be calculated and visualized in 3D.

An SSM can help understand and recognize normal variation over time in various populations. However, while this method captures a longitudinal, cross-sectional variation, applying this to an individual level is also possible. For example, given the 3D model of a bone at the age of 10 years, the SSM can predict the shape at 14 years by changing the variation described by the change in age and keeping other variations, describing person-specific characteristics, the same. In this way, a person-specific prediction of the anatomy can be made by applying an average effect. This prediction can help to identify pathological changes in practice. Such longitudinal predictions of skeletal anatomy can be valuable in tracking bone growth and supporting clinicians in diagnosing and treating bone abnormalities.

This scoping review provides an overview of the techniques and applications for spatiotemporal statistical shape modeling of skeletal structures during growth. We report state-of-the-art methods for constructing an SSM of developing bone and resulting statistical analyses focusing on the temporal aspect. The applications of an SSM are discussed along with the reported accuracy scores to investigate clinical feasibility.

## 2 Methods

We searched the PubMed and Scopus databases for studies in English until 20 February 2024. Searches were performed using the keywords ‘statistical shape model’, ‘spatiotemporal’ and ‘bone’ or associated terminology (see Table 1). We looked for additional relevant studies by snowballing, following references cited in the already identified studies, and added them to the synthesis.

**Table 1:** Search terms for literature search.

AND	AND	
Statistical shape model	Spatiotemporal	Bone
SSM	Longitudinal	Skeletal
Shape model	Development	
Shape analysis	Growth	
Morphology	Pediatric	
	Variation	
	Change	

The first author performed screening of title and abstract. Studies meeting the following criteria were included:

a. Involves the application of statistical shape modeling,
b. on a dataset comprising 3D skeletal structures,
c. in a pediatric population involving growth.

Articles were systematically searched for study characteristics such as anatomical region, dataset size and goal of the SSM. The model type, parametrization and alignment methods and shape analyses used to create the SSMs were extracted along with the reported variation explained by the first PCs. Outcomes regarding the application accuracy of the SSMs were collected, including prediction and correlation analyses if performed.

## 3 Results

### 3.1 Search results & study characteristics

Fourteen studies were identified for this review and included for data extraction (Table 2). Anatomical structures modeled in these studies included one or multiple lower limb bones, the mandible, the skull, and vertebrae. Most studies used a cross-sectional dataset of CT scans as a source for the 3D surface models (Carman et al., 2022; Coquerelle et al., 2011; Heutinck et al., 2021; Klop et al., 2021; Klop et al., 2024; Li et al., 2015; Li et al., 2018; McKinsey et al., 2023; Mercan et al., 2020; O'Sullivan et al., 2021; O'Sullivan et al., 2022; Peters et al., 2017; Sahlstedt, 2018). Only one study used a cross-sectional dataset of MRI scans (Shi et al., 2022). Three studies derived their bone models from post-mortem body CT scans (Carman et al., 2022; Klop et al., 2024; McKinsey et al., 2023). One study utilized scans of cadaveric mandibles (Klop et al., 2021), while the other studies sourced data from patient scans taken for clinical or research purposes (Coquerelle et al., 2011; Heutinck et al., 2021; Li et al., 2015; Li et al., 2018; Mercan et al., 2020; O'Sullivan et al., 2021; O'Sullivan et al., 2022; Peters et al., 2017; Sahlstedt, 2018; Shi et al., 2022). Age was the variable temporal factor in all included articles, varying between 0 and 2 months and 0 to 25 years. All studies aimed to generate a representative bone model for the temporal range covered in their dataset. The authors of six studies tested for correlation between shape-related variables and age (Coquerelle et al., 2011; Heutinck et al., 2021; Klop et al., 2021; Klop et al., 2024; Mercan et al., 2020; O'Sullivan et al., 2022). In seven articles, the predictive capability of the SSM was evaluated (Carman et al., 2022; Li et al., 2015; McKinsey et al., 2023; O'Sullivan et al., 2021; O'Sullivan et al., 2022; Sahlstedt, 2018; Shi et al., 2022). Two other studies focused on the ability to distinguish two groups based on shape data derived from the SSM (Coquerelle et al., 2011; Mercan et al., 2020).

**Table 2:** Characteristics and goal of included statistical shape models.

Study	Source	Bone	Dataset type & age range	Subjects (n)	Aim of the study
Carman et al., 2022	CT	Pelvis Femur Tibia/Fibula	CS 4–18 y	Pelvis: 331 Femur: 663 Tib/fib: 658	Understand shape development during growth. Predict new geometry using subject characteristics.
Coquerelle et al., 2011	CT	Mandible	CS 0–25 y	159	Differentiate between groups over time.
Heutinck et al., 2021	CT	Head	CS 0–2 y	65	Understand shape development and assess surgical outcome.
Klop et al., 2021	CT	Mandible	CS 1–12 y	874	Understand shape development during growth.
Klop et al., 2024	CT	Mandible	CS 0–22 y	678	Understand shape development during growth.
Li et al., 2015	CT	Skull	CS 0–3 y	56	Predict new geometry using subject characteristics.
Li et al., 2018	CT	Vertebrae	CS 3–10 y	30	Assess correlation between shape and age.
McKinsey et al., 2023	CT	Femur	CS 0–3 y	96	Predict new geometry using subject characteristics.
Mercan et al., 2020	CT	Skull	CS 0–6 m	Healthy: 117 Diseased: 81	Understand shape development during growth and differences between groups.
O'Sullivan et al., 2021	CT	Skull	CS 0–4 y	178	Create a representative bone model.
O'Sullivan et al., 2022	CT	Mandible	CS 0–4 y	242	Assess correlation between shape and age.
Peters et al., 2017	CT	Spine Vertebrae	CS 1–19 y	91	Assess correlation between shape/position and age.
Sahlstedt, 2018	CT	Proximal femur	CS 7–17 y	61	Create a representative bone model.
Shi et al., 2022	MRI	Lower limb	CS 6–19 y	Limb: 56 Pelvis: 29	Create a representative bone model.

Abbreviations: CS, cross-sectional; CT, Computed Tomography; FU, follow-up; L, longitudinal; m, month; MRI, Magnetic Resonance Imaging; y, year

### 3.2 Model type & parametrization

**Fig. 2:**
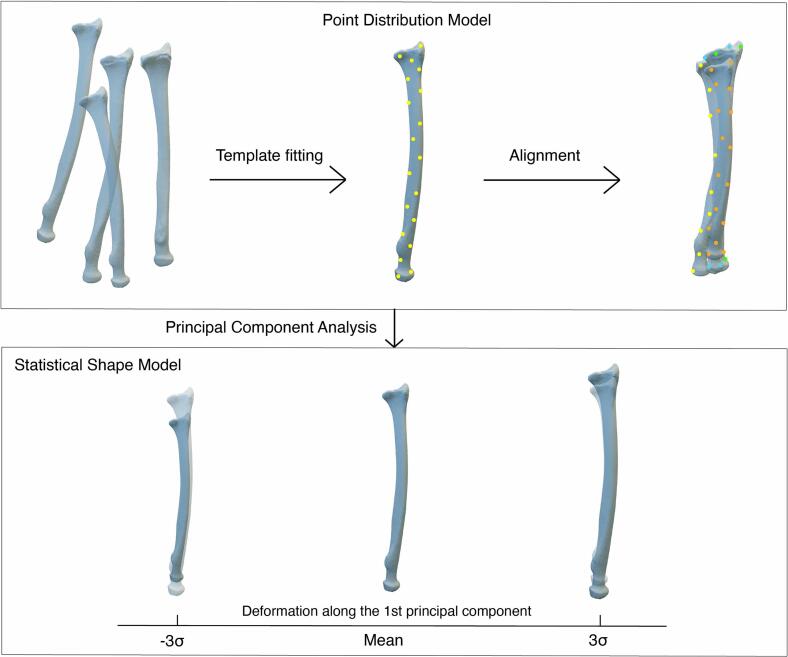
Visualization of the development of an SSM, with the radius as example. Shape correspondence among the dataset is established using template fitting of the Point Distribution Models (top). After alignment, PCA is applied to generate the SSM (bottom).

In most studies, a Point Distribution Model (PDM) was used to represent the model surfaces as data points in a three-dimensional coordinate system (Table 3) (Carman et al., 2022; Coquerelle et al., 2011; Klop et al., 2021; Klop et al., 2024; Li et al., 2015; Li et al., 2018; McKinsey et al., 2023; Mercan et al., 2020; O'Sullivan et al., 2021; O'Sullivan et al., 2022; Peters et al., 2017; Sahlstedt, 2018; Shi et al., 2022). A point cloud is created using anatomical landmarks, non-anatomical semi-landmarks, or evenly distributed surface nodes. Semi-landmarks are points that are positioned along curves or surfaces between traditional landmarks. Samples were fitted to a template to consistently sample data points among all subjects, known as parametrization (Fig. 2). Usually, the 3D model of the dataset’ mean or a sample close to the mean is used as a template, along with its surface representation by landmarks or evenly distributed surface points. Several techniques were applied to fit the PDMs to the template, including Radial Basis Functions, Thin-Plate Splines, Iterative Closest Point (ICP), and Coherent Point Drift (CPD) algorithms, as shown in Table 3. Four studies reported fitting errors to quantify the anatomical and/or geometric accuracy of the template fitting. Template-to-target registration using ICP and CPD algorithms resulted in average geometrical distances of 0.05 mm and submillimeter differences between the actual and expected landmark positions (Klop et al., 2021). The MeshMonk algorithm, a combination of rigid and non-rigid registration, resulted in a similar geometric accuracy of 0.05 mm. Anatomical validation showed a mean distance of 1.1 mm between the landmarks assigned by MeshMonk and by the observer (Klop et al., 2024). Sahlstedt (2018) reported mean distances between the registered volume meshes and the actual meshes of <0.2 mm and mean maximum distances in the order of 2 mm (Sahlstedt, 2018). Geometric fitting errors were low for all bones in Carman et al. (2022) after template fitting using radial basis functions (pelvis: 0.35 ± 0.08 mm, femur: 0.20 ± 0.04 mm, tibia/fibula: 0.16 ± 0.04 mm) (Carman et al., 2022).

**Table 3:** Techniques used to develop the Statistical Shape Models and resulting Principal Component scores.

Study	Model type	Parametrization	Alignment	Shape analysis	Principal Components # first principal components | % variation explained
Carman 2022	PDM - Surface nodes	Template fitting - Radial basis function	Rigid - Centre of mass	Standard PCA Procrustes PCA Scaled PCA	Standard PCA pelvis: 1 PC | 92 % Standard PCA femur: 1 PC | 98 % Standard PCA tibia/fibula: 1 PC | 97 % Procrustes PCA pelvis: 41 PCs | 90 % Procrustes PCA femur: 60 PCs | 90 % Procrustes PCA tibia/fibula: 39 PCs | 90 % Scaled PCA pelvis: 9 PCs | 90 % Scaled PCA femur: 36 PCs | 90 % Scaled PCA tibia/fibula: 15 PCs | 90 %
Coquerelle 2011	PDM - Anatomical landmarks - Semi-landmarks	Template fitting - Thin-plate spline	GPA	PCA	1 PC | 62 %
Heutinck 2021	Deformation model	Deformation	NR	PCA	1 PC | 67 %
Klop 2021	PDM - Semi-landmarks	Template fitting - ICP & CPD	GPA - Unscaled	PCA	1 PC | 78 %
Klop 2024	PDM - Surface nodes	Template fitting - Rigid & non-rigid ICP	Procrustes - Unscaled - Scaled	PCA	Unscaled: 1 PC | 92 % Unscaled: 3 PCs | 95 % Scaled: 24 PCs | 95 %
Li 2015	PDM - Anatomical landmarks - Semi-landmarks	Landmark registration	Rigid - Translation - Rotation	PCA	NR
Li 2018	PDM - Discrete points from curves	Curvature-based approach	Rigid - Anatomical coordinate system	PCA	NR
McKinsey 2023	PDM - Surface nodes - Anatomical landmarks	Template fitting - Radial basis function	Procrustes	PCA	1 PC | 99 %
Mercan 2020	PDM - Anatomical landmarks - Semi-landmarks	Template fitting - Thin plate splines	GPA - Unscaled	PCA	Healthy: 1 PC | 35 % SCS: 1 PC | 30 % 20 PCs | 90 %
O'Sullivan 2021	PDM - Anatomical landmarks	Template fitting - Non-rigid ICP	Procrustes	PCA	1 PC | 38 % 10 PCs | 90 %
O'Sullivan 2022	PDM - Anatomical landmarks - Semi-landmarks	Template fitting - Non-rigid ICP	Rigid - Landmarks - Unscaled	PCA	1 PC | 90 % 22 PCs | 99 %
Peters 2017	PDM - Anatomical landmarks	Template fitting - ICP	GPA - Scaled	Fitted equations - Scale maintained	
Sahlstedt 2018	PDM - Surface nodes	Template fitting - ICP and nonrigid registration	GPA - Scaled	PCA	1 PC | 33 % 23 PCs | 95 %
Shi 2022	PDM - Surface nodes	Template fitting - Radial basis function	Rigid - ICP - Scaled	PCA	Femur: 11 PCs | 75 % Pelvis: 9 PCs | 80 % Tibia: 9 PCs | 83 % Fibula: 5 PCs | 63 % Patella: 4 PCs | 43 %

Abbreviations: CPD, Coherent Point Drift; GPA, Generalized Procrustes Analysis; ICP, Iterative Closest Point; NR, Not Reported; PC, Principal Component; PCA, Principal Component Analysis; PDM, Point Distribution Model.

The two studies by Li et al. (2015, 2018) used different model types to parametrize the samples. In the first study, they identified landmarks across the surface and aligned them using rigid registration to a pre-set origin and orientation (Li et al., 2015). Their later study used a curvature-based approach to discretize surfaces into geometric curves and then align discrete points (Li et al., 2018). Heutinck et al. (2021) applied a deformation model using DEFORMETRICA, a framework to determine the population's average shape and its variations (Heutinck et al., 2021). The dataset is represented by a template and deformation vectors that can be used to warp the template to a subject's shape complex (Durrleman et al., 2014).

Summarized, the primary approach for this step in SSM development was the construction of a PDM with landmarks or surface nodes. Parametrization with template fitting was the most common, resulting in negligible registration errors.

### 3.3 Analysis of shape variation

Peters et al. (2017) analyzed shape variation of the vertebrae in relation to age by fitting equations to estimate landmark positions and rotation angles (Peters et al., 2017). Of all shape equations, 72 % displayed a significant age-dependent trend. All other studies used PCA to assess shape variation across the population (Table 3).

**Fig. 3:**
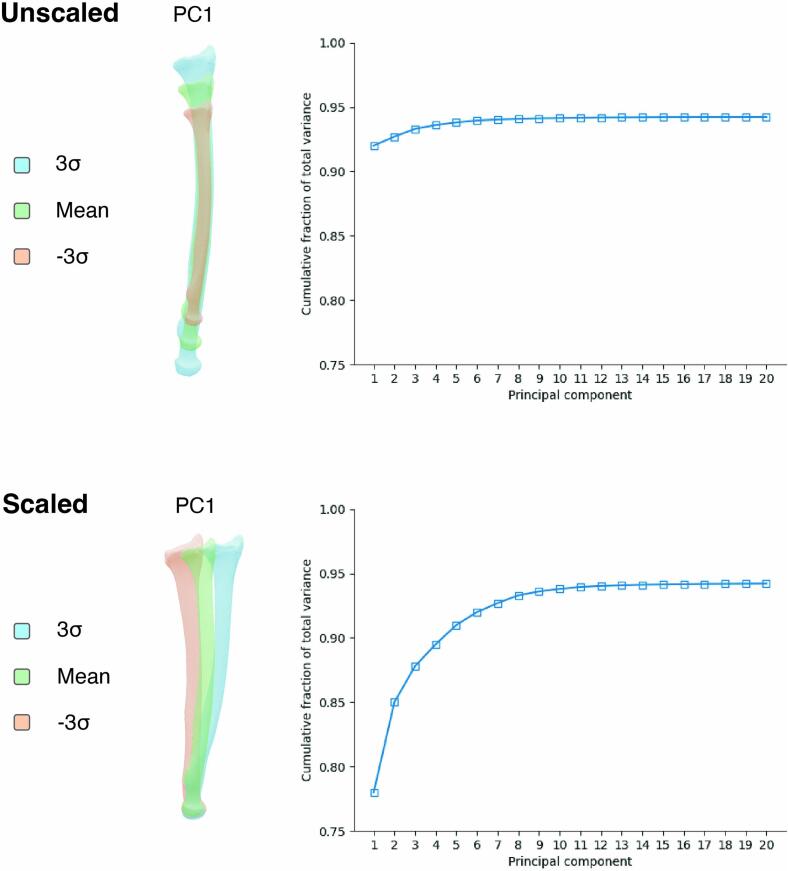
Visualization of the effect of scaling using synthetic data. Shape variation represented by the first shape at the left, and compactness plots at the right for (A) unscaled models and (B) scaled models.

Before conducting PCA, all samples must be aligned to achieve point correspondence as facilitated by the earlier parametrization step. In six studies, the differences in size were eliminated by scaling all samples to the mean size (volume) during the alignment process with Procrustes analysis (Coquerelle et al., 2011; McKinsey et al., 2023; O'Sullivan et al., 2021; Peters et al., 2017; Sahlstedt, 2018; Shi et al., 2022). This removed a large proportion of variation, especially in datasets involving growth, and revealed more subtle changes in the primary PCs. In six other studies, the scaling difference in samples across the temporal range was retained by applying rigid registration or Procrustes without scaling (Carman et al., 2022; Klop et al., 2021; Li et al., 2015; Li et al., 2018; Mercan et al., 2020; O'Sullivan et al., 2022). Incorporating size variation into the SSM is needed for accurately predicting new geometries, including bone size. In the second study of Klop et al. (2024) and the study of Carman et al. (2022) SSMs with and without scaling in the alignment or PCA were developed and compared (Carman et al., 2022; Klop et al., 2024). The first PC of the unscaled mandible SSM by Klop et al. (2024) explained 92 % of all variation, whereas the first PC of the scaled model accounted for only 67 % of the variation. For the scaled model, more PCs were required to cover 95 % of the variation (24 PCs compared to 3 for the unscaled model), making it a less compact model. The same trend was seen in Carman et al. (2022). Only one PC was needed to explain >90 % of the variation for the unscaled bone models. The SSMs constructed using Procrustes PCA, thus without size differences, used at least 40 PCs to describe 90 % of shape variation but revealed more detailed shape changes. These findings were consistent with the other studies: unscaled models were generally more compact than scaled SSMs (Fig. 3). The applied pipelines to develop an SSM, along with reported proportions of variation explained by the first PC(s), can be found in Table 3.

Recapitulating, stadium of growth, thus bone size, was the main variation across the dataset in most studies. Applying scaling mitigated this size variation, revealing more subtle differences in shape. The compactness of the SSMs increased when using unscaled samples; the first principal component(s) explained the majority of variation. Unscaled models were favorable when predicting new geometries, as size information was retained.

### 3.4 Correlation

In most studies, the first PC was dominated by age-related changes. In McKinsey et al. (2023), the first shape mode was greatly weighted by variation in the longitudinal plane, reflecting the change in femur length (McKinsey et al., 2023). In Mercan et al. (2020) and the first study of O'Sullivan et al. (2021), it was found that the first PC captured differences in overall skull size (Mercan et al., 2020; O'Sullivan et al., 2021). The latter reported a correlation between the first PC and age in the healthy and diseased populations. Heutinck et al. (2021) identified a correlation between the first shape mode and age as well, but also with cranial dimensions using Pearson's r correlation test (Heutinck et al., 2021). Three studies used Spearman's rank correlation test. They revealed that their first PCs correlated strongly with age, as shown in Table 4 (Klop et al., 2021; Klop et al., 2024; O'Sullivan et al., 2022) . Size difference was no longer a dominant factor in successive PCs, and no correlation with age was found (O'Sullivan et al., 2022). Klop et al. (2024) reported significant correlations to age for both the unscaled SSM and the rescaled model of the mandible (Klop et al., 2024). For the unscaled model, an increase in size was the most noticeable change of the first PC, but upon closer inspection, some additional age-related processes could be identified. In the rescaled SSM, the other age-related variations, like the gonial angle and chin protrusion, were more evident because size differences were absent. The same effect was observed in Carman et al. (2022) (Carman et al., 2022). The first PC of the standard model showed most of the variation, accounting for overall size. The first shape mode of the Procrustes PCA included more subtle growth-related changes in shape, like bone width and rotational factors.

**Table 4:** Correlations between shape features and temporal factors.

Study	Variable 1	Variable 2	Statistical test	Correlation coefficient, *p*-value
Heutinck 2021	PC1	Cranial width/height/length Age	Pearson's r	*r* = 0.75/0.84/0.84, p < 0.001 *r* = 0.76, p < 0.001
Klop 2021	PC1	Age	Spearman's r	*r* = 0.87, *p* < 0.001
Klop 2024	PC1 original PC1 scaled	Age	Spearman's r	r = 0.87, *p* < 0.0001 *r* = 0.82, p < 0.0001
Mercan 2020	PC1	Age healthy Age diseased	Linear regression	R^2^ = 0.68 R^2^ = 0.59
O'Sullivan 2022	PC1	Age	Spearman's r	*r* = 0.94, *p* < 0.01

In short, the first shape mode correlated strongly with the temporal factor age in all studies that investigated it. Also a scaled model, where size differences were excluded, showed a significant correlation between morphological features and age.

### 3.5 Model & prediction performance

The PCs can be used to reconstruct any shape in the dataset. The geometry is approximated as the mean shape plus a weighted sum of the included PCs, representing how much of the different modes of variation are present in the shape. In Sahlstedt, 23 of the 122 PCs were used to reconstruct training object shapes, leading to a mean RMSE, a measure for the deviation between the original and reconstructed shapes, of <0.5 mm (Sahlstedt, 2018). In the first study of O'Sullivan et al. (2021), a generalization error of 1 mm was found when including 15 PCs in the modeling of the skull. The error was <0.5 mm when all PCs were utilized, indicating that the model generalized well to unseen skull samples (O'Sullivan et al., 2021). Specificity values <0.7 mm demonstrated that novel skull instances generated by the SSM were also realistic. The model of O'Sullivan et al. (2022) generalized with an error < 0.5 mm when 15 PCs were used (O'Sullivan et al., 2022). The specificity error was 5.1 mm with 160 PCs. This could be attributed to the model size differences and the small sample size for model construction. The SSM of Shi et al. (2022) was used to reconstruct bones in a leave-one-out analysis (Shi et al., 2022). Reconstructing using a complete segmentation and the first PC as input yielded an RMSE of ±1 mm for all bone types. The authors tried to reconstruct bone geometries using a small set of landmarks as input as well. This resulted in slightly higher mean errors (<2 mm for the tibia, fibula and patella, 2.38 mm for the femur and 3.60 mm for the pelvis) but captured enough shape variance to reconstruct novel bone shapes accurately. This study also compared using an adult-based SSM to a pediatric SSM to reconstruct pediatric bones. The latter was significantly more accurate, supporting the value of a spatiotemporal population-specific SSM when modeling developing anatomy.

In their first study, Li et al. (2015) utilized a multivariate regression model to associate PC-scores, representing head geometry, with the predictors age and head circumference (Table 5) (Li et al., 2015). Based on those predictors, landmark prediction of fifteen unseen patients showed good accuracy when compared to the actual landmarks from CT data. Surface reconstruction, performed after fitting the template model, based on the predicted landmarks was reported acceptable. With a linear mixed model, age, head circumference and landmark locations were significant predictors of suture width and skull thickness. Hence, the authors expect the model to be useful for rapidly generating patient-specific finite element models using only a few predictors, such as age and head circumference. These predictors would be used to predict landmarks and geometric information, which in turn can be employed for morphing a template skull into a patient-specific model.

**Table 5:** SSM-based prediction models.

Study	Goal	Predictive model	Analysis	Predictive factors		R^2^	Error scores
Carman 2022	Predict new geometry - Inform clinical decisions - Comparison for children with abnormalities	PLSR	Leave-one-out	Bone measurements Age, height, mass, sex	Pelvis	0.976 (all factors) 0.951 (demographics)	RMSE [mm] 2.91 ± 0.99 (all factors) 3.23 ± 1.22 (demographics) Volume error [%] 9.90 ± 8.29 (all factors) 10.76 ± 9.18 (demographics)
					Femur	0.997 (height, femoral length) 0.970 (age, height)	RMSE [mm] 2.01 ± 0.62 (all factors) 2.72 ± 1.24 (demographics) Volume error [%] 8.62 ± 8.09 (all factors) 8.90 ± 9.20 (demographics)
					Tibia/ fibula	0.990 (height, tibial length) 0.966 (height, mass)	RMSE [mm] 1.85 ± 0.54 (all factors) 2.25 ± 0.96 (demographics) Volume error [%] 9.95 ± 9.86 (all factors) 11.17 ± 12.47 (demographics)
Li 2015	Predict new geometry - Generate patient-specific models - Curves to predict skull injury risk	Multivariate regression	Test set validation	Age & head circumference		NR	Mean error [mm] X: 0.07 ± 2.26 Y: 0.13 ± 3.05 Z: 0.93 ± 4.06
McKinsey 2023	Predict new geometry - Generate femur models for finite element analysis	PLSR	Test set validation	Age, height, weight		0.976	RMSE [mm] 1.97 Volume error [%] 10.1 ± 8.3
O'Sullivan 2021	Predict age based on bone shape	Linear regression	10-fold cross-validation	PCA shape vectors		0.770	RMSE [months] 6.1
O'Sullivan 2022	Predict age based on bone shape	PLSR	10-fold cross-validation	PLS shape modes		0.940	RMSE [months] 3.3

Abbreviations: NR, Not Reported; PCA, Principal Component Analysis; PLS, Partial Least Squares; PLSR, Partial Least Squares Regression; RMSE, Root Mean Square Error.

Both Carman et al. (2022) and McKinsey et al. (2023) used their SSM to predict individual lower limb bone geometries of new subjects based on predictive factors such as age, height and mass (Carman et al., 2022; McKinsey et al., 2023). With Partial Least Squares Regression (PLSR), the capacity of patient characteristics to predict PC scores was investigated (Table 5). McKinsey et al. (2023) chose age, height, and weight as predictors to estimate the first two PC scores. The estimated PC scores were used to generate predicted femur shapes of a test set, resulting in an overall RMSE of only 1.97 mm between the predicted and the actual models segmented from CT. Larger reconstruction errors were found at the metaphyses, with high morphological variation. Carman et al. (2022) performed a multiple comparison analysis between the first PC and predictive factors, including bone measurements and demographic factors (age, height, mass, and sex) to evaluate their predictive capacities. In the scaled SSM model, where bones were uniformly scaled by bone length, variations were not significantly explained by any of the predictive factors. This limited the ability of this model to predict new geometries. In the standard PCA model, the highest percentage of variation over all bones was explained by age, height, and bone measurements. The best set of predictive factors was determined using PLSR between all principal components and different combinations of predictive factors. The set of best-performing predictors was used in a leave-one-out analysis to predict weights for the PCs and reconstruct the geometry of the one ‘left-out’ subject. Lower RMSEs were found when bone length measurements and demographic factors were used for prediction. However, errors were still acceptable (±0.5 mm higher) when only demographics were used. The same applied to the volume errors, which were comparable to volumetric errors reported by McKinsey et al. (2023). For the femur and tibia/fibula, height and bone length measurements were the most important predictive factors. Age did not contribute to predicting long bone shapes as much as height and bone length. If bone measurements were unavailable, height and age were shown to be predictive values for the femur and height, along with mass for the tibia/fibula.

The other way around, O'Sullivan et al. (2021,2022) assessed if age could be predicted using skull and mandibular shape, respectively (O'Sullivan et al., 2021; O'Sullivan et al., 2022). In their first study, a linear age regression indicated that age in the range of zero to four years old can be inferred from skull shape with reasonable accuracy. In the latter study, age prediction was performed using PLSR analysis. The result was a significant correlation between predicted and true age.

In several studies, the effect of sex on bone shape was investigated, either as the predictor or the to-be-predicted variable. Coquerelle et al. (2011) assessed sexual dimorphism in the mandible from birth until adulthood, finding differences between males and females until 4 years old and after puberty (Coquerelle et al., 2011). Peters et al. (2017) found no sexual dimorphism in size, 3D shape, and orientation in their pediatric spine and vertebrae SSMs (Peters et al., 2017). Klop et al. (2024) showed a difference between sexes for four PCs and created a separate growth model for males and females (Klop et al., 2024). In O'Sullivan et al. (2021), a low correlation between sex and the shape modes was reported (O'Sullivan et al., 2021). Sex was found to be an important predictive factor for the pelvis but not for long bones in Carman et al. (Carman et al., 2022).

PLSR was successfully used in several studies for prediction based on an SSM. The first step in reconstructing a new subject's anatomy involves predicting the weightings of PCs. With those scores, the deviation from the average shape across different modes of variation is known, facilitating the reconstruction of the desired geometry. Height and, to a lesser extent, age, sex and mass are important predictive factors. Bone measurements can improve the prediction's accuracy but are less easy to obtain in clinical practice.

## 4 Discussion

Understanding changes in bone anatomy during growth and even predicting them is feasible with spatiotemporal statistical shape modeling. An SSM of a developing population is a valuable tool, not only for gaining insight into the growth process but also for differentiating normal from pathological growth, fostering the treatment of patients with osseous pathology.

Growth is a complex process about which little is known due to the scarcity of 3D scans of (healthy) children at different ages. Therefore, developing spatiotemporal SSMs of pediatric bones along the growth process is valuable.

Population size varied from tens to hundreds of subjects. A larger dataset with representative subjects helps to ensure all common variations are captured in detail within the SSM. However, quality of the samples should be a priority over the quantity. Carman et al. (2022) and Shi et al. (2022) found comparable reconstruction accuracy values, although the latter used a smaller dataset (±650 versus 56 subjects, respectively) to develop the SSM (Carman et al., 2022; Shi et al., 2022). This suggests additional training data may not always lead to a better-performing SSM. Especially when modeling pediatric anatomy with varying size, other factors may have a larger influence on the model's accuracy, such as the choice to apply scaling or not.

The origin and quality of the data used to develop the SSM should match the purpose of the model. Cadaveric subjects have the advantage of higher limits for radiation exposure and no moving artifacts, enhancing imaging quality. Scans of cadaveric bodies or bones suffice to explore the shape variation in a population, provided quality is good and uniform across the dataset and characteristics like age are available. For predicting individual anatomy, it is recommended to use the same type of training data as will be used to validate the model. In clinical practice, this would likely be CT scans of one anatomic region, with radiation exposure as low as reasonably possible. With the risk of moving artifacts, manual or semi-automatic segmentation is recommended to ensure high quality 3D models.

Bone models were, in most cases, parametrized using a PDM with (semi-)landmarks. A deformation model, as Heutinck et al. (2021) applied, is also a viable option (Heutinck et al., 2021; Gerig et al., 2016). However, the implicit representation of shape is less intuitive than explicit representations like PDMs, according to Adams et al. (2022) (Adams et al., 2022). PDMs are more easily interpreted and visualized, which is preferred for clinical application. Alignment using template fitting resulted in errors of <0.5 mm, which is in the same order of magnitude as a CT scan's spatial resolution. Assessing the anatomical accuracy of the template fitting is recommended if the PDM should represent the exact same anatomical location on every sample, for example for automatic landmark placement. However, anatomical accuracy does not have to be very high to capture the shape variation in a population. By assessing the geometric accuracy, one makes sure the PDM represents the bone surfaces well, which is sufficient to explore the shape variation reliably population-wise. Creating a PDM without landmarks is viable to capture all variations in the dataset, but alignment can be challenging. Parametrization guided by landmarks improves anatomical accuracy and is therefore recommended if available. Four studies used manually annotated landmarks during the development of the PDM (Li et al., 2015; McKinsey et al., 2023; Mercan et al., 2020; O'Sullivan et al., 2022). They checked the inter- and intra-observer variability of the landmarking process, which is advisable to ensure dense correspondence in the resulting PDM. Automatic landmarking, used in two studies, is recommended if a validated algorithm is available to avoid the time-consuming and error-prone annotation process (Coquerelle et al., 2011; Peters et al., 2017). The included studies were not consistent regarding the use of scaling during alignment. So, whether it is advisable to apply scaling depends on the goal of the SSM. Scaled models can reveal more subtle age-related shape variations. However, an unscaled SSM is advised for predicting anatomical shape, as the retained size information is essential to reconstruct anatomy accurately.

The reviewed studies consistently demonstrated that the most common shape variation, PC1, correlated strongly with age. Carman et al. (2022) and McKinsey et al. (2023) employed the demographic factors age, height, and mass to predict lower limb bone geometries (Carman et al., 2022; McKinsey et al., 2023). They achieved promising results in estimating PC scores and generating new bone shapes for unseen patients. Height was the most important predictor for the lower extremity; it is unknown if the same applies to other bones. In general, including more characteristics improves the prediction outcome. None of the included studies assessed the predictive value of ethnicity. However, that could be valuable as several studies have shown a relationship between ethnic origin and bone shape (Durbar, 2014; Zengin et al., 2016; Seeman, 1998) .

McKinsey et al. (2023) developed a well-performing model to predict lower limb geometries (Table 2) according to the RMSE and volume errors (McKinsey et al., 2023). However, mean and maximum nodal reconstruction errors were higher at the proximal and distal parts of the bone than along the shaft. Exactly those proximal and distal parts of the bones are most variable during growth, close to the joints and crucial to model with high accuracy. This would need improvement before using the model for clinical applications. In Carman et al. (2022), bone measurements contributed to the prediction accuracy. RMSEs were ± 0.5 mm lower than with only demographic predictive factors (Carman et al., 2022). It remains to be seen whether it is clinically relevant to conduct those bone measurements, if feasible at all without imaging, for a slightly smaller margin of error.

It is recommended to not only validate the model's accuracy to represent the population's bone shapes but also assess its feasibility for the clinical purpose. Otherwise, statistical shape modeling can appear as a black box for unfamiliar users, noted Johnson et al. (2023) in their review (Johnson et al., 2023). Appropriate outcome measures should be chosen to validate the model and its applications. Compactness, generality and specificity are common scores to describe an SSM's quality in general but are not clinically useable. Using a test set or leave-one-out analysis is advisable to validate prediction models by comparing the prediction with the original bone model. RMSE was reported in most included studies, but also the visualization of the error distribution across the model proved valuable in McKinsey et al. (2023) to get an understanding of the numbers (McKinsey et al., 2023).

The RMSE was the most reported outcome measure, which made it possible to compare the accuracy of the models, while the anatomical structure and validation method often differed. This made it difficult to compare the SSMs qualitatively. As described, proper validation aimed at clinical applicability is important to ensure the SSM's accuracy in representing anatomy at the population level. In addition, scores such as compactness, specificity, and generalization are preferred to be reported to assess the quality of the SSM, thus the method, in a general sense. For prediction, the RMSE is a general outcome measurement that enables a simple comparison between models. Other outcome measurements, such as bone length, angulation, and specific anatomical radiologic measurements, would provide more insight into applicability for a clinical case but make comparisons between models of different anatomy impossible.

In this review, we focused on modeling healthy growth. The clinical applicability of thoroughly understanding growth includes recognizing variability within the healthy range. It also allows for creating personalized references when the standard is unavailable. Predictions of healthy growth can aid in early recognition of deviations from the expected growth trend. When an extremity abnormality is diagnosed and becomes symptomatic, a corrective osteotomy might be the treatment of choice. Currently, the patient's contralateral healthy side is often used as ‘reference anatomy’ during pre-operative 3D planning. Scanning the contralateral side could become unnecessary when using an SSM-based prediction as a reference, reducing radiation exposure in children. Osteotomy planning in cases of bilateral abnormalities, where usually no reference anatomy is available, would also be enabled by an accurate prediction model for healthy anatomy (van Es et al., 2024). With these applications in mind, it is important to consider whether the prediction from the SSM is accurate enough for these goals. Outcome measures like RMSE are valuable for evaluating a model's technical accuracy in representing anatomical variations within a population. We recommend these technical metrics to enable model comparability while also emphasizing the need to explore clinically meaningful measures. Clinicians generally benefit from having a single, easy-to-interpret value that is directly applicable to clinical practice. Additionally, we encourage systematic data collection, including 3D models and demographic variables such as age, skeletal age, sex, and ethnicity. The completeness and quality of these data impact the model's ability to accurately capture group differences and predict 3D anatomical variations, enhancing clinical relevance.

This overview of state-of-the-art methods can be a foundation for future research into skeletal growth models using statistical shape modeling. In this study we chose for a scoping review method and a search strategy that allows for a broader exploration of the available literature on state-of-the-art applications of spatiotemporal shape modeling. It is a relatively new field of research; the number of publications is still small and heterogeneous. Finally, there was limited diversity in anatomical structures. Unfortunately, no studies on statistical shape modeling of the upper extremity were included. However, enhancing our understanding of anatomical averages and variations is also needed for those bones, particularly during growth.

In conclusion, this review aimed to bring together techniques and applications of spatiotemporal statistical shape modeling of 3D healthy skeletal structures, providing a starting point for future research. We demonstrated that spatiotemporal SSMs offer insights into anatomical variations during growth. It has the potential to predict skeletal geometry based on personal characteristics. If this also applies to patient-specific characteristics, these models could also become of value for diagnosing and preoperative planning in orthopedic procedures.

## CRediT authorship contribution statement

**Lily E. de Vries:** Writing – review & editing, Writing – original draft, Visualization, Methodology, Investigation, Formal analysis, Conceptualization. **Derek F.R. van Loon:** Writing – review & editing, Writing – original draft, Conceptualization. **Eline M. van Es:** Writing – review & editing, Supervision, Conceptualization. **DirkJan H.E.J. Veeger:** Writing – review & editing, Supervision, Conceptualization. **Joost W. Colaris:** Writing – review & editing, Supervision, Conceptualization.

## Declaration of competing interest

The authors have no professional or financial affiliations that may have biased the findings of this manuscript.

## Data Availability

No data was used for the research described in the article.
